# Astrocyte Support for Oligodendrocyte Differentiation can be Conveyed via Extracellular Vesicles but Diminishes with Age

**DOI:** 10.1038/s41598-020-57663-x

**Published:** 2020-01-21

**Authors:** Cory M. Willis, Alexandra M. Nicaise, Ernesto R. Bongarzone, Maria Givogri, Cory R. Reiter, Olivia Heintz, Evan R. Jellison, Pearl A. Sutter, Gregg TeHennepe, Guruprasad Ananda, Anthony T. Vella, Stephen J. Crocker

**Affiliations:** 1 Department of Neuroscience, University of Connecticut School of Medicine, Farmington, CT USA; 2 Department of Anatomy and Cell Biology, University of Illinois at Chicago, Chicago, IL USA; 3 Department of Immunology, University of Connecticut School of Medicine, Farmington, CT USA; 4 The Jackson Laboratory for Genomic Medicine, Farmington, CT USA

**Keywords:** Neuroscience, Glial biology, Astrocyte

## Abstract

The aging brain is associated with significant changes in physiology that alter the tissue microenvironment of the central nervous system (CNS). In the aged CNS, increased demyelination has been associated with astrocyte hypertrophy and aging has been implicated as a basis for these pathological changes. Aging tissues accumulate chronic cellular stress, which can lead to the development of a pro-inflammatory phenotype that can be associated with cellular senescence. Herein, we provide evidence that astrocytes aged in culture develop a spontaneous pro-inflammatory and senescence-like phenotype. We found that extracellular vesicles (EVs) from young astrocyte were sufficient to convey support for oligodendrocyte differentiation while this support was lost by EVs from aged astrocytes. Importantly, the negative influence of culture age on astrocytes, and their cognate EVs, could be countered by treatment with rapamycin. Comparative proteomic analysis of EVs from young and aged astrocytes revealed peptide repertoires unique to each age. Taken together, these findings provide new information on the contribution of EVs as potent mediators by which astrocytes can extert changing influence in either the disease or aged brain.

## Introduction

Astrocytes are an abundant and critical cell type for neural development including synapse formation and axon myelination, and ensuring homeostasis of the adult central nervous system (CNS)^1^. In many neurodegenerative diseases, inflammatory responses by glial cells represent a major component associated with neuropathological changes. Specifically, astrogliosis is a prominent feature found in every chronic neurodegenerative disease and aging^2,3^. Therefore, it is not unexpected that impaired astrocyte functions are increasingly identified as contributing features to the pathogenesis of many chronic neurological diseases^4^, including many age-related neurodegenerative diseases such as Alzheimer’s^5–7^, Parkinson’s^8^, and amyotrophic lateral sclerosis (ALS)^9,10^. A specific indicator of perturbed astrocytic function may be the loss of myelin maintenance support and reduced oligodendrocyte integrity which are notable changes in aging and further exacerbated by disease^4^.

Age is the single largest risk factor for the development of neurodegenerative diseases^11^. Numerous transcriptomic and epigenetic studies have identified significant changes in the expression of glial-specific genes associated with normal aging^12,13^. Natural aging is also associated with the development of a CNS pro-inflammatory status that is attributed to the development of cellular senescence predominantly among glial cells^14^. As a biological process, cellular senescence can be induced by a wide-range of cellular stressors including oxidative stress, inflammation, intracellular accumulation of DNA damage, and metabolic dysfunction^15–21^. The contribution of oxidative, metabolic, and inflammatory stressors have been implicated in aging and an accumulation of cells exhibiting cellular senescence-like changes have also been found to accumulate with greater frequency than in healthy aging^22,23^. Accordingly, an increasing number of studies have been investigating the influence of age and the processes of cellular senescence as contributing to changes associated with the pathology of several prominent degenerative diseases^24^.

Paradoxically, senescent cells often comprise only a minority of cells within even very old tissues^25–29^, therefore it is becoming more apparent that senescent cells can drive age-related diseases in a non-cell autonomous fashion. Reactive astrocytes have been implicated as a common and deleterious cell response in neurological diseases and astrocytes have also been identified a cellular population liable to develop senescence in some disease states^23,30^. Hence, in the present study we sought to determine if aging of primary cultures of astrocytes would induce development of a senescent phenotype. Accordingly, we characterized the impact of aging on the secretory phenotype of astrocytes and measured the functional impact of these changes as alterations in support of oligodendrocyte maturation. We identify extracellular vesicles (EVs) as important and sufficient components of the astrocyte secretome which communicate a negative effect of cellular senescence as a loss of astrocytic support for OPC maturation. Additionally, proteomic analyses revealed significant changes to the cargo of EVs from aged astrocytes, which could be suppressed by treatment with rapamycin, a compound known to have senescence suppression properties^31–33^. Taken together, these data demonstrate a novel paradigm wherein astrocytes aged *in vitro* develop a senescence-like phenotype that is accompanied by alterations in the effect of EVs on the propensity of astrocytes to support OPC differentiation. These findings have implications for understanding the basis for astrocyte phenotypes with aging and their influences on CNS functions.

## Results

### Astrocytes maintained in culture long-term are pro-inflammatory and express senescence-like changes

**Figure 1:**
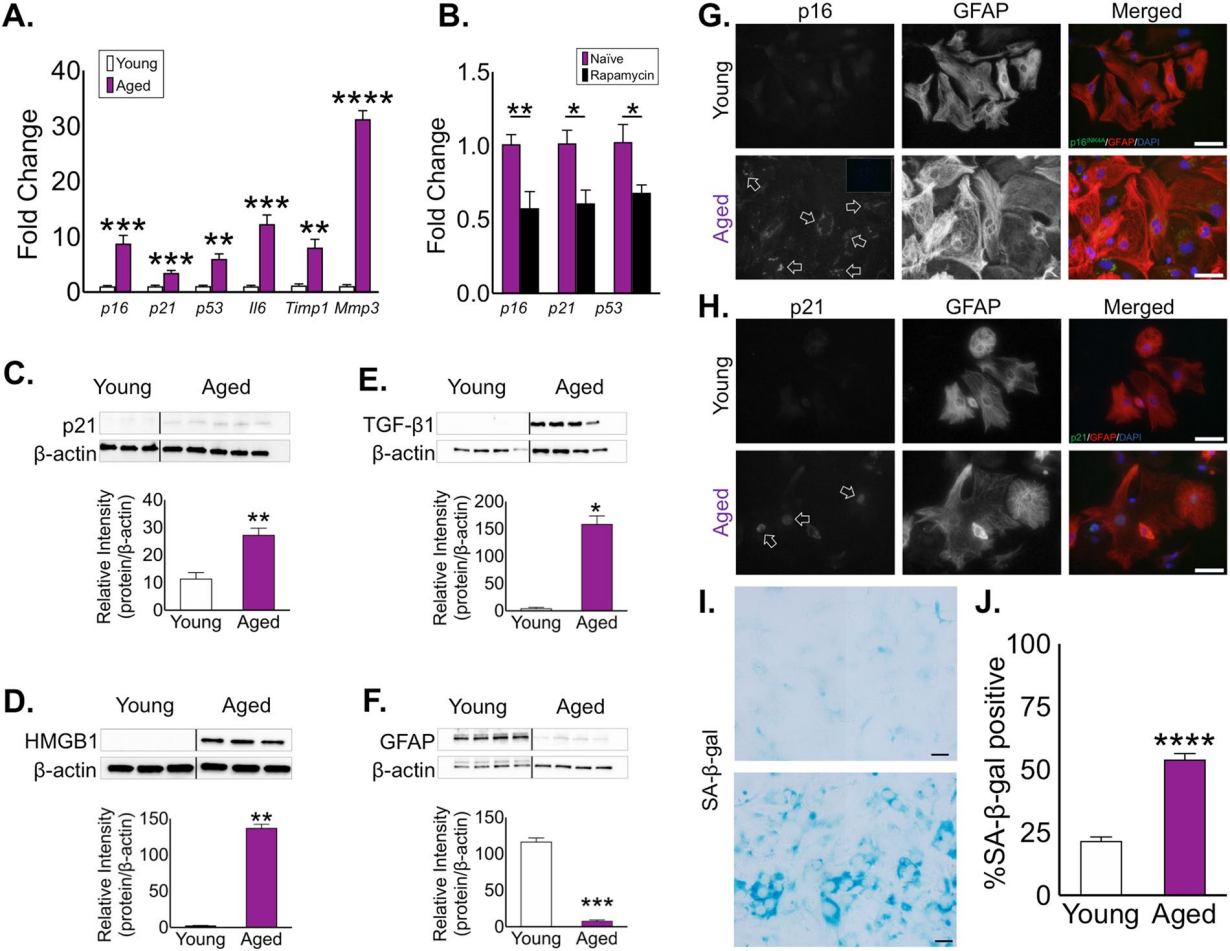
Astrocytes aged *in vitro* develop a senescence-like phenotype. (**A**) Analysis of mRNA expression for the senescence-associated genes *p16*^*INK4A*^, *p21*, *p53*, *Il-6*, *Timp-1*, and *Mmp-3* by qPCR in young (white) and aged (purple) astrocyte cultures. (**B**) Expression of senescence-related genes p16^INK4A^, p21, and p53 following rapamycin treatment (25 nM/day, 72 h) Fold expression determined by normalization to expression in young astrocytes. Western blot analyses of (**C**) p21, (**D**) HMGB1, (**E**) TGFB1 and the intermediate filament protein (**F**) GFAP from young and aged astrocyte cell lysates. Densitometry (a.u.) for each factor was used to determine expression relative to β-actin. Representative immunocytochemistry for (**G**) p16^INK4A^ and (**H**) p21 in young and aged astrocytes. Scale bar, 20 μm. (**I**) Representative SA-β-gal staining of young and aged astrocyte cultures, and (**J)** quantification of SA-β-gal staining in quadruplicate independent cultures. Scale bar, 20 μm. (**I**) Significance as indicated where: (**A**) **P < 0.01, ***P < 0.001, and ****P < 0.0001, Student’s t-test with Welch’s correction. (**B**) *P < 0.05, **P < 0.01, Student’s t-test with Welch’s correction. (**C–F**) *P < 0.05, **P < 0.01, and ***P < 0.001, Student’s t-test with Welch’s correction. (**J**) ****P < 0.0001, Student’s t-test with Welch’s correction. Values are expressed as mean ± SEM.

To determine how length of  time in culture with minimal passages affected astrocytes we maintained primary murine astrocyte cultures (with weekly media changes) for either ≤4 weeks (young) or ≥16 weeks (aged). To limit the potential confounding effects of cell division, re-plating or splitting of these cultures, each was kept with minimal manipulations to avoid induction of a replicative senescence phenotype^34,35^. We then collected these cultures and analyzed for differences in established markers of cellular senescence. We first performed an analysis of genes known to be associated with aging and cellular senescence by qPCR analysis of mRNA from young and aged astrocytes. This analysis revealed higher expression of *p16*^*INK4A*^, *p21*, and *p53*, as well as pro-inflammatory SASP factors *Il-6*, *Mmp-3*, and elevated expression of the astrocyte activation marker, *Timp-1*, in the aged astrocyte cultures (Fig. 1A). Several of these genes are associated with cellular senescence, a cellular phenotype most often linked to acquisition of a pro-inflammatory state and cell cycle arrest. Indeed, disrupted cell cycling can result from increased expression of cyclin dependent kinase inhibitors *p16*^*INK4A*^ ^36,37^, *p21*^37^, and the tumor suppressor gene, *p53*^38^. We then performed an analysis of astrocyte-specific genes known to be dysregulated with aged and identified an increase in the water channel *Aqp4* and a decrease in *S100β* with no change in *Il-4* (Supplementary Fig. 1A–C). *Aqp4* up-regulation in our aged cultures is consistent with previous reports^12,39^. Flow cytometry analysis of young and aged astrocytes revealed a significant increase of cells in the G1 phase and a significant decrease in the proportion of cells in the G2/S phase in aged astrocyte cultures when compared with young cultures (Supplementary Fig. 2A–C)^40^. An additional analysis of cellular proliferation using proliferating cell nuclear antigen (PCNA) revealed a biomodal distribution of GFAP + cells in the aged cultures although there were no quantitative differences in over all cellular proliferation between young and aged cultures (Supplementary Fig. 3). To examine whether these changes were related to cellular senescence, we treated cultures of aged astrocytes with rapamycin, a macrolide compound which has gained popularity for its effectiveness in the field of aging as a means to suppress aspects of the senescent state^41^. Treatment of aged cultures with rapamycin (12.5 nM−37.5 nM/day, 72 h) significantly reduced expression of p16^INK4A^, p21, and p53 (Fig. 1B). This effect of rapamycin on these genes in aged astrocytes was also found to be concentration dependent (Supplementary Fig. 4A–C).

Immunoblotting was then performed using cell lysates which confirmed an increased production of the senescent marker p21 in the aged cultures (Fig. 1C). We next analyzed the expression of pro-inflammatory factors associated with aging including high mobility group box 1 (HMGB1) (Fig. 1D) and transforming growth factor beta (TGF-β) (Fig. 1E). These were also found to be elevated in aged astrocytes. Interestingly, GFAP expression in the aged astrocytes was reduced when compared to the expression in young cultures (Fig. 1F). This is consistent with previous reports identifying reduced GFAP in aged rodents and in human astrocytes exposed to a specific senescence-inducing agent^41,42^. We confirmed the cellular expression of p16^INK4A^ and p21 in the aged astrocytes by immunofluorescent cytochemistry while only a minimal signal for either markers was detected in young astrocytes (Fig. 1G,H). Further, a significant increase in the sub-cellular localization of HMGB1 to the cytosol was observed in aged astrocytes compared to young astrocytes (Supplementary Fig. 5A–C); a shift that is consistent with previous reports^43–45^.

Lastly, we assessed senescence associated (SA)-β-gal activity, which catalyzes the hydrolysis of β-galactosides into monosaccharides that occurs in cells exhibiting senescent-like changes was present in the aged astrocyte cultures^25^. A readily observable and quantifiable increase in SA-β-gal activity was found in the aged cultures but not the young astrocyte cultures (Fig. 1I,J). Together, these data confirmed the development of a senescence-like phenotype in aged primary astrocyte cultures.

### EVs from aged astrocytes do not support OPC maturation

**Figure 2:**
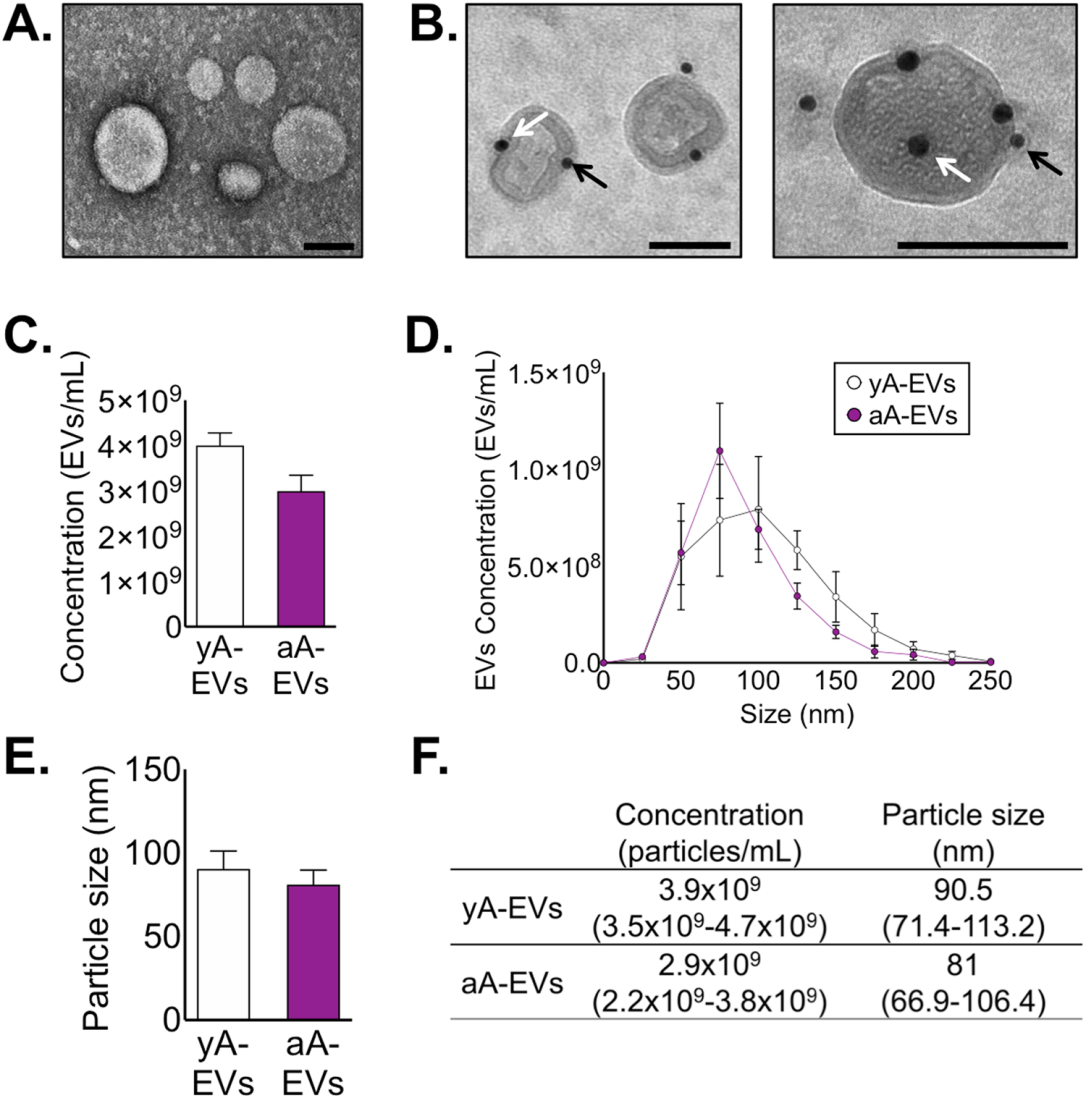
Identification and characterization of extracellular vesicles from young and aged astrocyte cultures. (**A**) Negative stain electron micrograph of EVs isolated from ACM of cultured astrocytes. (**B**) Electron micrographs of astrocyte-derived EVs in ACM verified by immunogold electron microscopy against the EV marker TSG101 and astrocyte marker GFAP. White arrowheads indicate 15 nm GFAP gold particles and black arrowheads indicate 10 nm TSG101 gold particles. Scale bar, 100 nm. Nanoparticle tracking analysis of ACM: particle concentration (**C**), particle size distribution (**D**), and mean particle size (**E**) in ACM of young (open circles) and aged astrocytes (filled circles). (**F**) Comparative table with particle concentration and mean particle size. Significance as indicated where: (**C,E**) *P* > 0.05, Student’s t-test with Welch’s correction. (**D**) *P* > 0.05, two-way ANOVA, Bonferroni’s post-hoc test). Values are expressed as mean ± SEM.

SASP factors released from senescent cells can exert a compelling influence on the other cells in a tissue by modifying the extracellular microenvironment, which can lead to loss of homeostatic control and even tissue deterioration associated with aging^46^. Emerging evidence now points to a contributing role for EVs as a major component of the SASP^47^. We have previously shown that young cultured astrocytes release EVs, which are readily identifiable by the astrocyte marker GFAP^48^. To test whether EVs were released from aged astrocytes in culture, we isolated EVs from the conditioned media of young and aged astrocytes and assessed the presence of EVs by negative staining and transmission electron microscopy (EM). This revealed that comparably shaped EVs were released from both young and aged astrocytes in culture and all EVs were within the size range of 50 to 150 nm (Fig. 2A). To confirm the identity of these EVs, and not rogue cellular debris, we performed immuno-EM staining for the EV marker TSG101 (black arrows) in conjunction with the astrocyte marker GFAP (white arrows) (Fig. 2B). Particle tracking analyses (nanosight) were also performed to determine whether EV release was affected by culture age. We found that EVs were released from both young and aged astrocytes (yA-EVs and aA-EVs, respectively) and the proportions did not differ in concentration (Fig. 2C) or size distribution (Fig. 2D,E). Together, these findings confirmed that EVs are released from astrocytes even after protracted time *in vitro* and that a cellular senescence phenotype did not negatively impact EV release.

**Figure 3:**
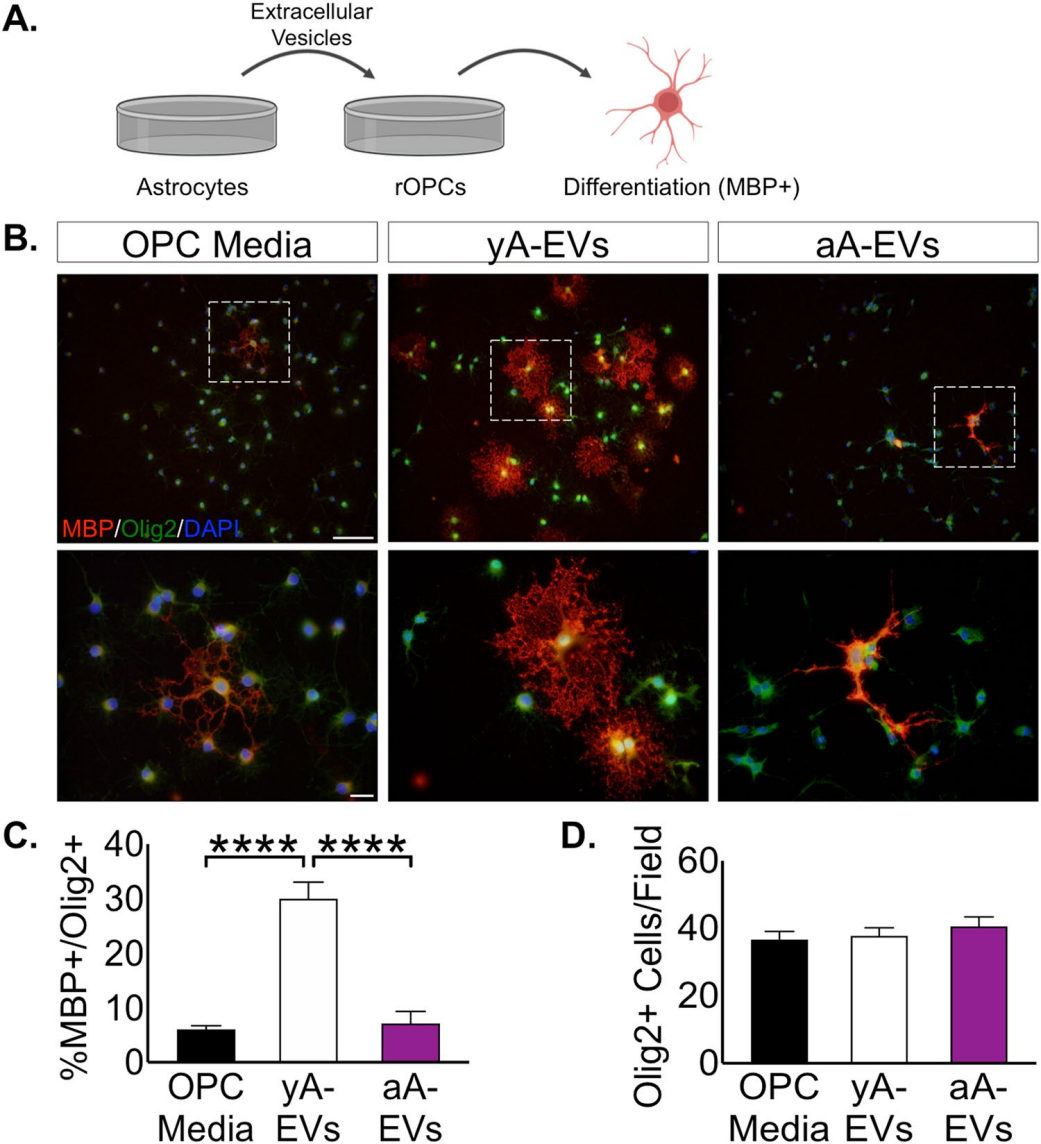
Extracellular vesicles from aged astrocytes do not support oligodendrocyte differentiation. (**A**) Experimental design used to test the effect of EVs isolated from ACM of young or aged astrocyte cultures on OPC maturation (rOPCs: rat OPCs). ACM from astrocytes was collected after 48 h, EVs isolated, and applied to rOPCs. Differentiation of OPCs was assayed after 48 h. (**B**) Representative images of mature oligodendrocytes (MBP+/OLIG2+) resulting from either young or aged EVs. Scale bar, 100 μm. Magnified panel scale bar, 20 μm. (C) Quantification of OL maturation following young and aged EV treatment. (D) Quantification of number of OLIG2+ OPCs under each treatment condition. n = 7–11 independent cultures per treatment. Significance as indicated where: (**C**) ****P < 0.001, one-way ANOVA, Tukey’s multiple comparisons test). yA-EVs = young astrocyte EVs; aA-EVs = aged astrocyte EVs. Values are expressed as mean ± SEM. Image of oligodendrocyte in Panel A from BioRender.com.

We next sought to evaluate the function of EVs from young and aged astrocytes using a physiologically relevant functional measure of oligodendrocyte progenitor cell (OPC) differentiation. We selected OPC differentiation because it is well established that astrocytes are critical for oligodendrocyte survival and CNS myelination during development^49–51^. Astrocytes are also a source of trophic support for the maintenance of myelin integrity^52^. Lastly, white matter loss occurs in healthy aging which is associated with changes in astrocytes to a more inflammatory phenotype^53^. These lines of reasoning provided the impetus to determine whether aged-astrocyte EVs (aA-EVs) differed from young astrocyte (yA-EVs) in terms of a measurable impact on OPC differentiation. To test this idea, primary rat OPCs (rOPCs) were treated with purified EVs (48 h) isolated from young or aged astrocyte conditioned media (ACM) and then the maturation of oligodendrocytes (OLs) was assessed by immunocytochemistry for the mature myelin protein, myelin basic protein (MBP) (Fig. 3A). Application of yA-EVs was found to induce robust maturation of MBP+/OLIG2 + OLs, whereas application of aA-EVs failed to promote any successful maturation of OLs in these cultures (Fig. 3B,C). Importantly, the number of OLIG2+ cells did not differ between these conditions indicating that the failure of maturation was not a result of increased cell death of OPCs (Fig. 3D). Thus, EVs from aged astrocytes exhibited a reduced propensity to support OPC maturation.

We then wanted to determine if these EV-mediated signaling effects are unique to aged astrocytes or were they a common phenomenon that arises with astrocytic dysfunction resulting from inflammation? To assess this possibility, young and aged astrocyte cultures were pre-treated with the pro-inflammatory cytokines IL-1β (10 ng/mL/day, 48 h) or IFNγ (10 ng/mL/day, 48 h), EVs isolated from the ACM and applied to naive cultures of rOPCs (Supplementary Fig. 6A). Application of EVs from IL-1β treated young astrocytes was found to significantly reduce the successful maturation of OLs compared to untreated yA-EVs, whereas application of EVs from IL-1β or IFNγ treated aged astrocytes did not significantly change the maturation of OLs when compared to untreated aA-EVs (Supplementary Fig. 6B,C). As we had found in the previous set of experiments, the number of total OLIG2+ cells did not differ between the conditions (Supplementary Fig. 6D). Thus, cytokine-induced dysfunction in young astrocytes reduces the ability of EVs to support OPC maturation, whereas the reduced propensity to support OPC maturation of aA-EVs are unique to the aged phenotype.

### EVs from aged astrocytes have an altered proteome

**Figure 4:**
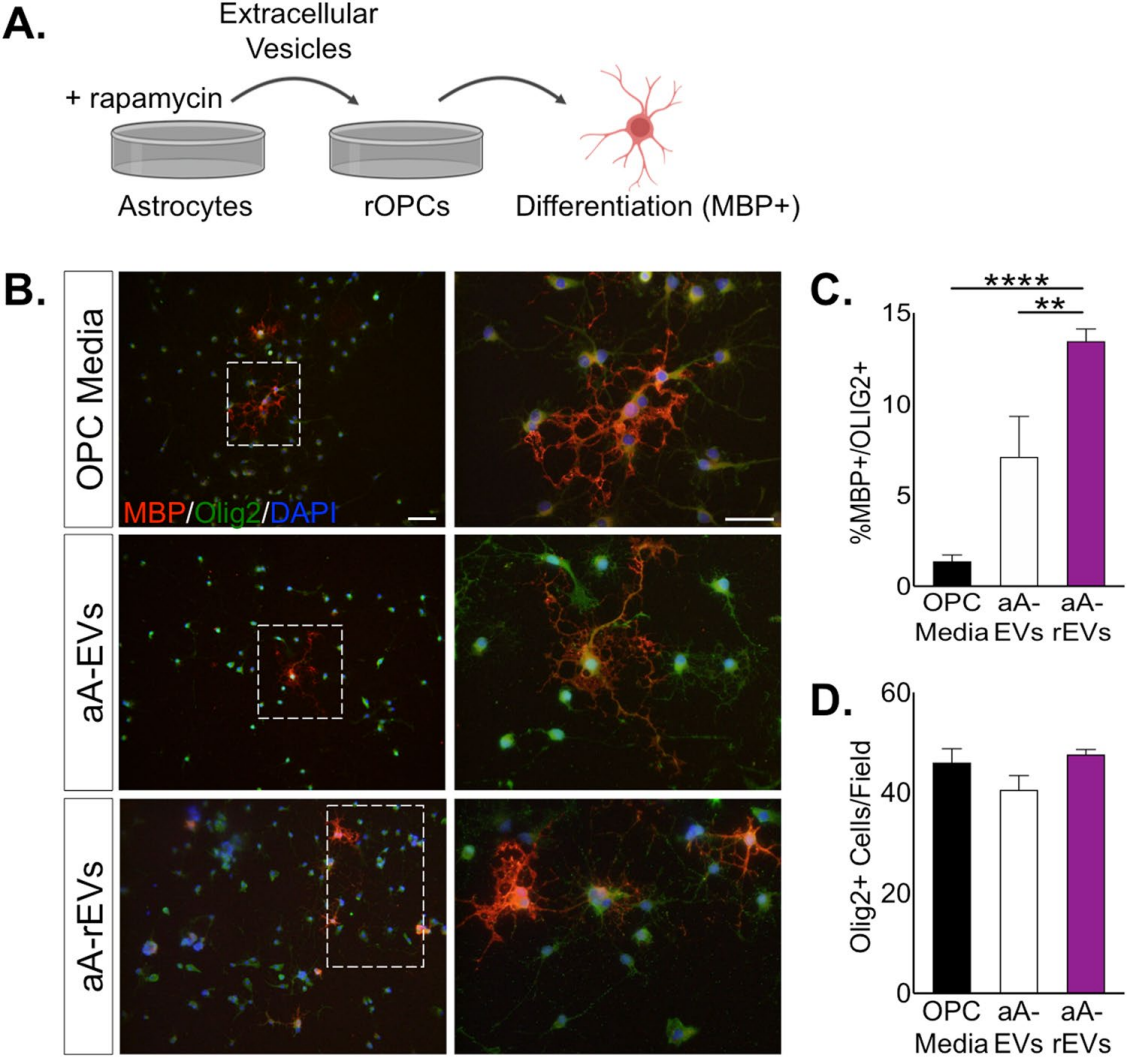
Negative effect of EVs from aged astrocytes is suppressed by treatment with rapamycin. (**A**) Experimental design to test whether pre-treatment of aged astrocytes with rapamycin (25 nM/day) for three days improved the ability of EVs to support OPC maturation (rOPCs, rat OPCs). ACM from rapamycin-treated astrocytes was collected after 48 h, EVs isolated and applied to rOPCs. Differentiation of OPCs was assayed after 48 h. (**B**) Representative images of mature oligodendrocytes (MBP+/OLIG2+) resulting from either aged or rapamycin-treated aged EVs. Scale bar, 100 μm. Magnified panel scale bar, 20 μm. (**C**) Quantification of OL maturation following aged and rapamycin-treated aged EV treatment. (**D**) No differences in number of OLIG2+ cells were observed from the varying treatments on the OPCs. n = 7–12 independent cultures per treatment. Significance as indicated where: (**C**) ****P < 0.001, one-way ANOVA, Tukey’s multiple comparisons test. aA-EVs = aged astrocyte EVs; aA-REVs = aged astrocyte rapamycin EVs. Values are expressed as mean ± SEM. Image of oligodendrocyte in Panel A from BioRender.com.

To determine whether the attenuated expression of senescence associated genes with rapamycin treatment of aged astrocytes improved the functional ability of aged EVs to support OPC maturation, we pre-treated cultures of aged astrocytes with rapamycin (25 nM/day, 72 h), collected the EVs from the ACM and applied these to naive cultures of rOPCs (Fig. 4A). Analysis of OL maturation revealed that when compared with EVs from control-treated aged astrocyte cultures, application of EVs from rapamycin-treated aged astrocytes significantly increased the differentiation of rOPCs into mature MBP+/OLIG2+ OLs (Fig. 4B,C). As we had found in the previous set of experiments, the number of total OLIG2+ cells did not differ between the conditions while the proportion of mature OLs was increased (Fig. 4D).

To interrogate the basis for the effect of aging and rapamycin on EV functions, we profiled the peptide content of EVs from young, aged, and rapamycin-treated aged astrocytes by mass spectrometry^54^. A total list of 1199 mouse proteins were identified and each protein was assigned a protein identification probability score. We retained 656 proteins that had a high protein identification probability (≥0.75). Of the 656 proteins identified, 25 were uniquely expressed in young astrocytes, while 47 were unique to aged astrocytes (Supplementary Fig. 7A). Over two-thirds of all identified proteins (69.2%) was annotated as belonging to extracellular exosomes (Gene ontology GO:0070062, false discovery rate (FDR) = 3.8E^−240^), with 21 identified proteins (3.1%) belonging to extracellular vesicles (Gene ontology GO:1903561, FDR = 3.0E^−14^).

**Figure 5:**
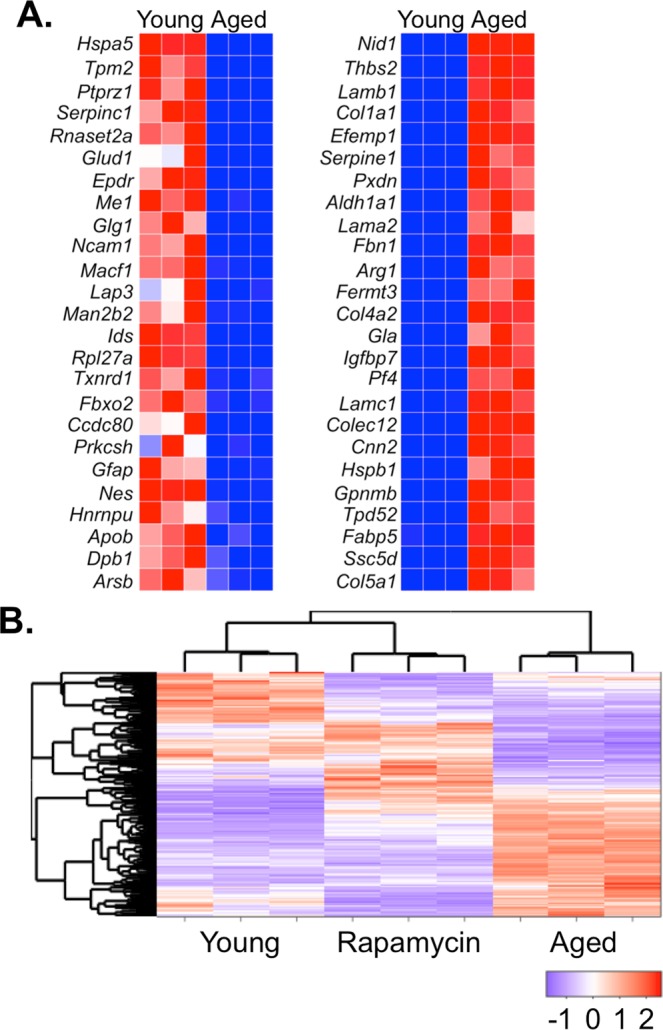
Proteomic analysis of young and aged astrocyte EV proteome. (**A**) Top 25 differentially expressed proteins (as listed by gene name) following quantitative analysis of protein abundance between young and aged astrocyte-derived EVs. (**B**) Differential expression and hierarchical cluster analysis between replicates using protein-wise exact tests. An FDR cutoff of 0.01 was used to identify significantly differentially expressed proteins.

Differential expression analysis using protein-wise exact tests for differences was performed and a FDR cut-off of 0.01 was used to identify significantly differentially expressed proteins. To examine more closely the specific changes between EVs from young and aged astrocytes, we generated heatmaps of the top 25 expressed EV proteins which revealed robust and distinct differences between these populations (Fig. 5A). We next examined the impact of rapamycin on aged astrocytes as reflected in the EV proteome which identified 275 proteins that were differentially expressed in at least one of the three comparisons. From this we performed a hierarchical clustering of these proteins and protein-wise centering and scaling of counts was performed to enable comparison across samples (Fig. 5B). Results of the analyses of the counts of the differentially expressed protein sets clearly separated the three groups; with the rapamycin-treated aged astrocyte EVs appearing to more closely resemble the proteome make-up of EVs from young astrocytes than aged. When EVs from aged astrocyte cultures were compared with EVs from rapamycin-treated aged astrocyte cultures we found 86 peptides enriched to treated EVs and 94 peptides that were no longer detected (Supplementary Fig. 7B). When compared with EVs from young astrocytes in culture, EVs from rapamycin-treated aged astrocytes we identified 74 peptides that were uniquely enriched and 47 that were not found in EVs from younger cultures (Supplementary Fig. 7C). Hence, while the clustering analysis suggested that the proteome of rapamycin-treated aged astrocytes were more similar to young than aged, the proteome of EVs after rapamycin treatment from aged cultures acquired distinct and unique differences from either naive young or aged cultures.

Together, our data analysis of the proteomic content of EVs from the three different groups suggests an aging protein signature of EVs from aged astrocytes that can be modified following treatment with rapamycin, which supports a role for therapies focused on reducing senescence to improve cellular and EV function in aged astrocytes.

## Discussion

In this report we have demonstrated that primary murine astrocytes when cultured long-term, and with low-passage number, develop a distinct phenotype from younger cultures that is represented in the effects of their EVs on oligodendrocytes. Unique to this study, we identified EVs as a critical component of the aged phenotype and characterized an altered proteome which had a functional impact on inhibiting the maturation of OLs from OPCs. We have also determined that the aging of primary murine astrocytes in this setting was amenable to therapeutic intervention as we had shown that rapamycin was effective in suppressing the transcriptional and functional phenotypes of aging-related changes in these cells.

Study of cellular aging is a rapidly growing field that has translated into clinical trials to affect the cellular senescent phenotype in cancer, idiopathic pulmonary fibrosis, and osteoarthritis^55^. Age has long been recognized as a process limiting the myelinating potential of the CNS^56^. Within the CNS, astrocytes are an abundant and heterogenous cell population that are known to be necessary for a wide variety of processes vital to CNS functioning^57^. Although astrocytes have been implicated as drivers of disease progression in several neurodegenerative diseases, the functional impact of aging on astrocytes and their consequence on myelination has not been previously assessed. Our findings, therefore, may indicate an important pathological component of the aging process which may induce processes related to senescent-associated genes in astrocytes as significant contributors to the loss of regenerative potential in many of these diseases. We have now determined that astrocytes aged in culture display many genes associated with cellular senescence, including: SA-β gal staining and increased *p16*^*INK4A*^, *p21, and p53*. *p16*^*INK4A*^, *p21, and p53* are cyclin-dependent kinase inhibitors. These hallmarks are often used to identify senescent cells that exhibit mitotic arrest and many of these genes can play a central role in the pro-inflammatory component of senescent cells^29,30,38,58^. For instance, increased expression of senescence associated cell cycle genes are thought to act through the retinoblastoma (Rb) pathway, which inhibits the action of cyclin-dependent kinases, in turn suppressing the expression of proliferation-associated genes^59–61^. Up-regulation of these genes can also result in chromatin reorganization, which results in changes in expression or regulation of genes related to inflammation and oxidation as well as other inflammatory mediators^55,62^. Previous studies have applied exogenous stressors, such as H_2_O_2_, as a means by which to induce premature senescence in primary cells^63^. Our results demonstrate that long-term culturing of primary astrocytes results in spontaneous development of aged-related changes and can result in the expression of cellular senescence-associated genes. While the effects of rapamycin to decrease age-related increases in gene expression were correlated with differences in the impact of aA-EVs on oligodendrocyte maturation, the regulation of mTOR by rapamycin are not limited to cellular senescence and thus translation of these findings to cellular senescence *in vivo* will require additional study.

Another key finding of this study was our identification of EVs as key contributors to the cellular effects of the inflammatory phenotype of aged cells. This is consistent with growing awareness that soluble factors which comprise the SASP from senescent cells which also implicate EVs and can serve as a major functional component of the astrocyte secretome^47^. Functional characterization of the SASP is important in determining the relevance of potential paracrine effects since EVs are critical mediators of intercellular communication in health and disease^64,65^.

Our proteomic analyses of EVs, which revealed significant changes in proteins with long-term culturing of astrocytes, may contribute to our understanding of how age of astrocytes can elicit functional differences from EVs. In terms of the impact of astrocytic EVs on OPC maturation, we found that EVs from young astrocytes had protein tyrosine phosphatase zeta (PTPRZ), which has been shown to be critical not only for the maturation of OLs from OPCs^66^ but also in functional recovery from demyelinating lesions in multiple sclerosis (MS)^67^. This might suggest that EVs from young but not aged astrocytes are capable of protein transfer into OPCs which contributes to their increased propensity to support OPC maturation^68^. In contrast, proteins identified in the EVs from aged astrocytes revealed changes in metabolism and the extracellular environment which may be distinct from cellular profiles^69^ but are consistent with the theme that ECM dysregulation is associated with aging and related diseases^70^. For instance, Arginase-1 was a candidate senescence-related factor identified in our proteomic analysis of EVs from aged astrocytes that was not present in EVs from young astrocytes. Arginase-1 is a ureohydrolase in the urea cycle that catalyzes arginine to ornithine and urea. In terms of its CNS functions, Arg1 is perhaps best known as an anti-inflammatory gene expressed by microglia and macrophages and has been used as an indictor of a regenerative capacity of these cells^71,72^. Analysis of Arg1 expression across neural cell types indicates homogenous expression while our data would indicate that aging *in vitro* results in elevated production by astrocytes. Elevated production of Arg1 could reflect altered uric acid metabolism, which has been reported to be elevated with aging which can have beneficial and detrimental consequences. Uric acid functions as an antioxidant levels have been suggested to correlate with improved cognitive function in aged individuals, and neuroprotection in certain conditions, such as ischemic brain injury and Parkinson’s^73,74^. In demyelinating diseases, such as MS, reduced uric acid are correlated with disease progression^75^. Hence, astrocyte production of Arg1 may reflect compensatory age (time) dependent changes in metabolism. However, Arg1 would not likely represent a direct mediator of oligodendrocyte growth arrest we observed in our study, and work by others has also reported reduced neuroprotective potential of astrocytes aged *in vitro*^76^. Instead, Arg1 in aged astrocyte EVs may reflect an overall increase in oxidative stress with aging where Arg1 would foster uric acid production as a means to scavenge elevated peroxynitrite levels^76,77^. Whether these specific mechanisms and changes in Arg1 associated with astrocyte EVs *in vitro* correlate with functional differences in astrocytes and changes in brain chemistry with aging remains to be tested.

Among the peptides found to be most up-regulated in the EVs from our long-term astrocyte cultures, no obvious known inhibitors of OPC maturation were identified. This suggests that it is likely the composite effect of multiple factors together rather than a single factor that is most important for why EVs from aged astrocytes do not support OPC maturation. Additionally, the cytokine HGMB1 was found to be associated with EVs from both young and aged astrocytes, with a significant increase in abundance in aA-EVs that was capable of being suppressed following rapamycin treatment (Supplementary Fig. 8A,B). This is consistent with published findings revealing a p53-dependent release of HGMB1 as a component of the senescence phenotype^43^ and supports our own findings of increased *p53* gene expression in aged astrocytes (Fig. 1A). We also recognize and should point out that EVs contain not only proteins, but also mRNAs, miRNAs, and cytoplasmic DNA^78^. Since no obvious players in the regulation of OPC differentiation were identified in our proteomics analysis, one could argue that non-peptide mechanisms, such as miRNA, could represent a more plausible mechanism by which aging in astrocyte EVs imparts effects on other cells. Nevertheless, future studies to interrogate the complex associations and functional relationships of various proteins, miRNA, RNA in EVs from young and aged astrocytes would be warranted to resolve these questions and potentially provide additional insights. In the context of understanding the astrocytic component of disease, further studies will need to be performed to determine if dysregulation of these proteins in astrocytes is a result of the normal aging process in astrocytes or induction of senescence.

In our studies we also found that expression of the senescence genes *p16*^*INK4A*^, *p21, and p53* in aged astrocytes could be effectively suppressed by rapamycin, and that treatment with this agent resulted in a functional improvement in the effect of EVs from aged astrocytes to enhance OPC maturation. This treatment effect indicates that mTOR activity was increased in senescent astrocytes and that affecting this pathway can impact the influence of senescence phenotypes on other cell types. We found that inhibition of mTOR signaling in aged astrocytes (Supplementary Fig. 9A,B) was associated with a changes in EV functions that then resembled the effects of EVs observed from younger astrocytes in terms of their impact on oligodendrocytes (Fig. 4B,C). These data suggest that in the aged astrocytes, signaling downstream of mTOR may function as a potent regulator of the EV proteome. Indeed, our proteomic data suggest striking differences in EV peptides, which reflect the chronologic age of astrocytes. Future interrogation of how mTOR signaling manages the EV proteome may be of interest to a wide range of neurological conditions for which astrocytic EVs have been implicated or could be inferred with aging.

Age is recognized as the single biggest unmodifiable risk factor for the development of neurodegenerative diseases among which Alzheimer’s and Parkinson’s diseases are considered the two most common^24,79,80^. Growing evidence supports a role for astrocyte senescence in driving disease progression and worsening in these patients^81,82^. Our observations introduce a salient question on the contribution of aging to reactive astrogliosis. Reactive gliosis, and astrocytosis, is not a binary state of activation, but rather, it encompasses a spectrum of functional states that can have beneficial or detrimental influences on the resolution of neuroinflammation^3,83–85^. We propose that age confers a potentially powerful influence on the intrinsic and extrinsic changes that can occur in astrocytes throughout natural passage of time and/or modified via disease-related stressors. Future interrogation of these could be useful to identify and corroborate similarities observed between astrocytes in the aged brain and astrocytes in disease and serve to distinguish normal healthy aging in astrocytes from the etiology of neuropathology. Moreover, the contribution of astrocyte EVs as prognostic, diagnostic, or therapeutically relevant mediators for any disease is currently unknown.

Our study may provide a basis upon which the development of senescence in aging in astrocytes could be used to distinguish from abnormal aging or disease. It is tempting to speculate that disease-associated aging of astrocytes typified by cellular senescence contributes to a reduced ability of astrocytes to support the normative functioning of the CNS: clinically, this may be represented by fostering neural dysfunction contributing to cognitive decline among certain individuals of advanced age or suffering from age-related disease^6,12,13^. Observations in the aging human brain have identified the presence of aged astrocytes and reported senescence in astrocytes by evidence of increased expression of *p16*^*INK4A*^, a cell-cycle regulator and hallmark of senescence, and matrix metalloproteinase 3 (*MMP3*), a common factor associated with the SASP^30^. *In vitro* experiments have supported the notion that astrocytes can induce a senescence program. Human and rodent astrocytes subjected to chronic reactive oxygen species (ROS) can undergo replicative senescence, characterized by the presence of SA-β-gal activity, growth arrest, and increased expression of the cell-cycle regulators p16^INK4A^ and p21^63^. Our data provide an additional perspective that many of the stress-related changes can be evoked by long-term culture without requiring growth arrest. We also implicate EV-mediated intercellular communication as a process reflective of the aging process that may be involved in dissemination of the influence of these stress and age-related changes in astrocytes onto other cell types in the aged brain.

In summary, we have determined that long-term culture of astrocytes impacts their phenotype that is reflected in their EVs. This provides a new perspective on the role of aging on astrocytes and EVs in health and disease. These data also suggest the possibility that aging negatively effects the ability of astrocytes to provide homeostatic support as evidenced by the lack of OL maturation from OPCs following treatment by EVs from senescent astrocytes. Delineating free SASP factors and those carried by EVs will be an important distinction in identifying overrepresented pathways to develop potential therapeutics. Cell-specific targeting of cellular senescence in astrocytes and the impact of senotherapies on astrocytes in preventing age-related CNS diseases could represent future means by which to restore the natural ability of astrocytes to maintain the normal functioning of the CNS. With the expanding awareness of the diverse critical homeostatic functions of astrocytes, understanding how these cells adopt a senescent phenotype and how this contributes to disease may represent a practical therapeutic approach to diminish and/or delay the impact of aging as a limiting force on health-span and regenerative potential in the CNS.

## Materials and Methods

### Primary glial cultures and treatments

All procedures involving animals in this study were only conducted with prior approval from the Institutional Animal Care and Use Committee (IACUC) at the University of Connecticut School of Medicine and in accordance with guidelines set forth by the National Research Council of the National Academies Guide for the Care and Use of Laboratory Animals. Cultures were generated from cerebral cortices of neonatal C57BL/6 mouse pups (P0-P3) using a neural tissue dissociation kit (Miltenyi Biotec, Cambridge, MA, USA), as previously described^48,86,87^ and consistent with previous reports of 90–97% GFAP + cells^88^. Culture flasks were subjected to weekly media changes (Dulbecco’s modified eagle medium, 1% Pen-strep, and 10% heat-inactivated fetal bovine serum). Primary cultures were established and maintained for either a short timeframe ≤4 weeks *in vitro* (“young”) or for an extended timeframe ≥12 weeks *in vitro* (“aged”). Once cultures reached age for experimentation, they were replated: cells were then trypsinized, pelleted, re-plated and then allowed to expand to approximately 80% confluence prior for treatment. Treatment with rapamycin used increasing concentrations (12.5 nM, 25 nM, 37.5 nM/day/72 h). Following treatment cells were collected in TriReagent and RNA was isolated for qPCR analysis.

### Immunocytochemistry

Astrocytes were plated (3 × 10^4^ cells) on laminin-coated coverglass (50 μg/mL), fixed in 100% ice-cold methanol, permeabilized using triton-x100 and then blocking buffer was added (10% normal goat serum). Cells were then incubated with primary antisera overnight (Supplementary Table 2). Immunoreactivity was visualized using fluorescent conjugated secondary antisera and counterstained with 4′,6-diamidino-2-phenylindole (DAPI) to identify nuclei. Immunostaining was analyzed using image analysis software (Olympus IX71 microscope with CellSens Software, Olympus, Tokyo, JP).

### Cellular RFI quantification

Cellular HMGB1 quantification was performed using ‘open composite image’ and ImageJ software (NIH). Three color fluorescent Images of HMGB1/GFAP/DAPI were split into 3 channels. The borders of a single astrocyte were obtained by freehand selection of the GFAP signal of an independent astrocyte within the GFAP channel, then GFAP fluorescence intensity was measured and then the same selection was applied to the HMGB1 channel. Raw fluorescence intensity for both channels was measured for the same area of a single astrocyte. The area and integrated density values for each selected area were recorded and repeated for multiple cells per treatment (technical replicates) and from independent experiments (biological replicates). The following equation was used to obtain a corrected total cell fluorescence [CTCF] from the raw intensity of each channel: CTCF = Integrated Density − (Area of selected cell × Mean fluorescence of background readings). CTCF values were then used to establish the HMGB1:GFAP ratio for each cell and for comparisons between treatment groups.

### Cell cycle analysis

Young and aged astrocytes (1 × 10^6^ cells) were re-suspended in PBS and fixed (70% ethanol/30 mins/4 °C) and washed with PBS. Cells were pelleted and re-suspended in DAPI staining solution (1 μg/mL DAPI in PBS) and then analyzed on an LSRII flow cytometer (Becton Dickinson). Cell cycle data was analyzed using MODFitLT software using at least 20 l events/sample (Verity Software, Topsham, ME, USA). Cell cycle phases were modeled assuming diploid cells and one cell cycle. Gaussian curves were fit to G0/G1, S, and G2/M phases to allow for calculation of the percent of cells in each phase. Averages for the percentage of cells in each phase were the result of three independent experiments per condition.

### Senescence β-galactosidase staining

β-galactosidase staining was performed on astrocytes (50,000 cells/coverglass) according to the manufacturer’s instructions (Cell Signaling Technology, Danvers, MA, USA). Images were captured at 20x using identical phase contrast settings (Olympus IX71, CellSens Software, Olympus, Tokyo, JP).

### Quantitative real-time polymerase chain reaction (qRT-PCR)

Total RNA from cultured astrocytes and converted into cDNA via reverse transcription (iScript cDNA synthesis kit, BioRad, Hercules, CA, USA), according to the manufacturer’s protocol as described previously^88^. Synthesized cDNA samples were amplified for qRT-PCR using mouse primer pairs for specific senescence markers (Supplementary Table 1) using the SsoAdvanced Universal SYBR Green Supermix (BioRad, Hercules, CA, USA) according to the manufacturer’s protocol. Analyses were performed on a BioRad CFX96 Touch Real-Time PCR Detection System. Relative expression of mRNA was calculated using β-actin to establish comparative cycle threshold analyses, as previously described^89^.

### Western blot

Western blot analysis was performed as previously described^48^. Briefly, astrocytes were lysed in RIPA buffer (with protease inhibitor cocktail) and separated by SDS-PAGE. Proteins were transferred to nitrocellulose and immunoblotted using unconjugated antisera against p21, GFAP, TGF-β, HMGB1, mTOR, or phospho-mTOR (Supplementary Table 2) and visualized using HRP-conjugated secondary antisera and chemiluminescence. Full blots included in Supplementary Fig. 10A–D.

### Extracellular vesicle generation and isolation

To generate EVs, astrocytes were cultured and let grow to 80% confluence, media was removed, cells washed 3x with phosphate buffered saline (PBS [0.01 M]), and serum-free media (DMEM supplemented with 1% Pen Strep) was added. After 48 h, EVs were isolated from conditioned serum-free media, as previously described^48^ and EV pellets were re-suspended in 200 μl of sterile, 0.22 μm-filtered PBS. Astrocytes were then treated with either IL-1β (10 ng/mL), IFNγ (100 ng/mL), or 25 nM of rapamycin once per day for 48 h (cytokines) or 72 h (rapamycin) in serum-free media. ACM was then collected and EVs isolated.

### Electron microscopy and immunogold labeling

Negative staining and Immunogold labeling were performed using 15 μl drops of EVs, as previously described^48^. Immunogold antibodies and concentrations included in Supplementary Table 2. Grids were imaged using a transmission electron microscope (TEM; Hitachi H-7650).

### Nanosight analysis

Purified EVs were analyzed on a NanoSight NS300 instrument equipped with a 488 nm laser (Malvern Instruments, United Kingdom). This equipment undergoes regular calibrations with Nanosight polystyrene latex calibration beads to confirm instrument accuracy. Collection methods were optimized following parameters as previously described^90^. EV samples were diluted (1:25) in PBS and injected using a motorized syringe pump speed of 100. Video recordings of 60 s repeated five times were collected for each sample, with consistent collection parameters of 30 fps, camera level 16, slider shutter 1300, and slider gain 512. Data were analyzed by NTA 3.2 software (Malvern Instruments, United Kingdom) and distribution data was binned into sizes by 10 nm in GraphPad Prism.

### Primary rat oligodendrocyte cultures and EV treatment

OPCs were obtained from the cerebral cortices of neonatal rat pups (postnatal day 0–2) plated on poly-l-ornithine coated coverslips, as previously described^91^. EVs were isolated as described above and OPCs were treated with 50 μl of an EV suspension containing an equivalent number of EVs (1.999 × 10^8^ particles) as determined from the Nanosight analysis from either naive young or aged astrocytes, IL-β or IFNγ pre-treated young or aged astrocytes, or rapamycin-treated aged astrocytes for 48 h and then fixed and stained for OLIG2 and MBP (Supplementary Table 2).For all experiments, four to five fields of view at 20x using identical image capture settings were assessed by an experimenter blinded to treatments (Olympus IX71, CellSens Software, Olympus). For analysis of OPC differentiation, all OLIG2+ cells and MBP+ cells were counted and the percentage of MBP+ cells calculated. All EV-treatment experiments were performed using quadruplicate biological replicates and repeated at least twice using separately derived cultures.

### In solution protein digestion

Extracellular vesicle pellets were resuspended in 1XRIPA buffer containing Halt Protease Inhibitor Cocktail (ThermoFisher Scientific, Waltham, MA, USA), and then homogenized by sonication and cleared by centrifugation (14,000 rpm, 4 °C, 10 min). Proteins were extracted by Chloroform/Methanol precipitation using established protocols. Protein pellets were dissolved and denatured (8 M urea, 0.4 M ammonium bicarbonate, pH 8) and then reduced by addition of 1/10 volume of 45 mM dithiothreitol and incubated (37 °C/20 min), and then alkylated with the addition of 1/10 volume of 100 mM iodoacetamide with incubation in the dark (RT/ 20 min). The urea concentration was adjusted to 2 M by the addition of water prior to enzymatic digestion at 37 °C with LysC for 4 h, then trypsinized (Seq. Grade Mod. Trypsin 16 h). Protease:protein ratios were estimated at 1:50. Samples were acidified by the addition of 20% trifluoroacetic acid, then desalted using C18 MacroSpin columns (The Nest Group, Southborough, MA, USA) following the manufacturer’s directions with peptides eluted with 0.1% TFA, 80% acetonitrile. Eluted sample was speedvaced dry and dissolved in MS loading buffer (2% aceotonitrile, 0.2% trifluoroacetic acid). A nanodrop measurement (Thermo Scientific Nanodrop 2000 UV-Vis Spectrophotometer) determined protein concentrations (A260/A280). Each sample was then further diluted with MS loading buffer to 0.08 µg/µl, with 0.4 ug (5 µl) injected for LC-MS/MS analysis.

### LC-MS/MS on the Thermo Scientific Q Exactive Plus

LC-MS/MS analysis was performed on a Thermo Scientific Q Exactive Plus with a Waters nanoAcquity UPLC system, using a Waters Symmetry® C18 180 µm × 20 mm trap column and a ACQUITY UPLC PST (BEH) C18 nanoACQUITY Column 1.7 µm, 75 µm × 250 mm (37 °C) for peptide separation. Trapping was done at 5 µl/min, 97% Buffer A (100% water, 0.1% formic acid) for 3 min. Peptide separation was performed at 330 nl/min with Buffer A: 100% water, 0.1% formic acid and Buffer B: 100% acetonitrile, 0.1% formic acid. A linear gradient (90 minutes) was run with 3% buffer B at initial conditions; 5% B at 1 minute; 35% B at 50 minutes; 50% B at 60 minutes; 90% B at 65–70; and back to initial conditions at 71 minutes. MS was acquired in profile mode over the 300–1,700 m/z range using 1 microscan; 70,000 resolution; AGC target of 3E6; and a full max ion time of 45 ms. MS/MS was acquired in centroid mode using 1 microscan; 17,500 resolution; AGC target of 1E5; full max IT of 100 ms; 1.7 m/z isolation window; normalized collision energy of 28; and 200–2,000 m/z scan range. Up to 20 MS/MS were collected per MS scan on species with an intensity threshold of 2E4, charge states 2–6, peptide match preferred, and dynamic exclusion set to 20 seconds.

### Peptide identification

Data was analyzed using Proteome Discoverer software v2.2. Data searching was performed using the Mascot algorithm (version 2.6.0) against the SwissProtein database with taxonomy restricted Homo sapiens. The search parameters included tryptic peptides with up to 3 missed cleavages, 10 ppm precursor mass tolerance and 0.02 Da fragment mass tolerance, and variable (dynamic) modifications of methionine oxidation, carbamidomethylated cysteine, deamidated asparagine or glutamine. Normal and decoy database searches were run, with the confidence level set to 95% (P < 0.05). Scaffold Proteome Software_version 4.8.7 was used to validate MS/MS based peptide and protein identifications. Peptide identifications were accepted if they could be established at greater than 95.0% probability by the Scaffold Local FDR algorithm. Protein identifications were accepted if they could be established at greater than 99.0% probability and contained at least 2 identified peptides.

### Statistical analyses

Data were analyzed using either a Welch’s t-test, one-way ANOVA with Tukey’s multiple comparisons test or two-way ANOVA with Bonferroni’s multiple comparisons test, where appropriate and as indicated, using GraphPad Prism version 7 for Mac OS X. Differences were considered significant when P < 0.05. Data are presented as mean ± SEM.

## Supplementary information

Supplemental Figures and Tables.

## Data Availability

Data deposition: The mass spectrometry proteomics data have been deposited to the ProteomeXchange Consortium via the PRIDE partner repository, https://www.ebi.ac.uk/pride/ (dataset identifier PXD017058).
