# Supplementary material for: Astrocyte Support for Oligodendrocyte Differentiation can be Conveyed via Extracellular Vesicles but Diminishes with Age
Source: Sci Rep. 2020 Jan 21;10:828. doi: 10.1038/s41598-020-57663-x (PMC6972737; doi:10.1038/s41598-020-57663-x)
Supplement: Supplementary file 1 — Supplemental Figures and Tables. [file 41598_2020_57663_MOESM1_ESM.pdf]

Astrocyte Support for Oligodendrocyte Differentiation can be Conveyed via Extracellular Vesicles but Diminishes with Age

Cory M. Willis[1], Alexandra M. Nicaise[1], Ernesto R. Bongarzone[2], Maria Givogri[2], Cory R. Reiter[2], Olivia Heintz[1], Evan R. Jellison[3], Pearl A. Sutter[1], Gregg TeHennepe[4], Guruprasad Ananda[4], Anthony T. Vella[3], Stephen J. Crocker[1]*

Departments of Neuroscience[1] and Immunology[3]
University of Connecticut School of Medicine, Farmington, CT;
[2]Department of Anatomy and Cell Biology, University of Illinois at Chicago, Chicago, IL;
[4]The Jackson Laboratory for Genomic Medicine, Farmington, CT

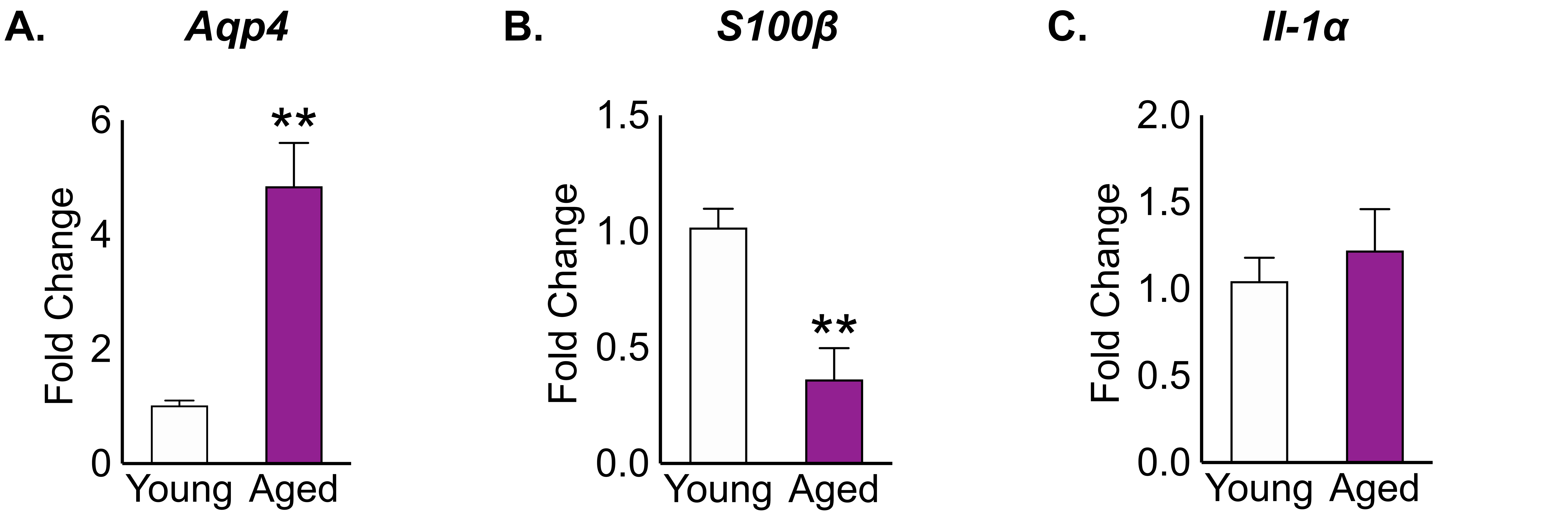

Supplementary Figure 1. Astrocyte specific gene analysis following long-term culturing. Analysis of mRNA expression for astrocyte-specific genes (**A**) *Aqp4*, **(B**) *S100β*, and (**C**) *Il-1α* in young (white) and aged (purple) astrocytes. Fold expression determined by normalization to *β-action* expression in young astrocytes. (**A-C**) *P < 0.05, **P < 0.01, Student's t-test with Welch's correction. Values are expressed as mean ± SEM.

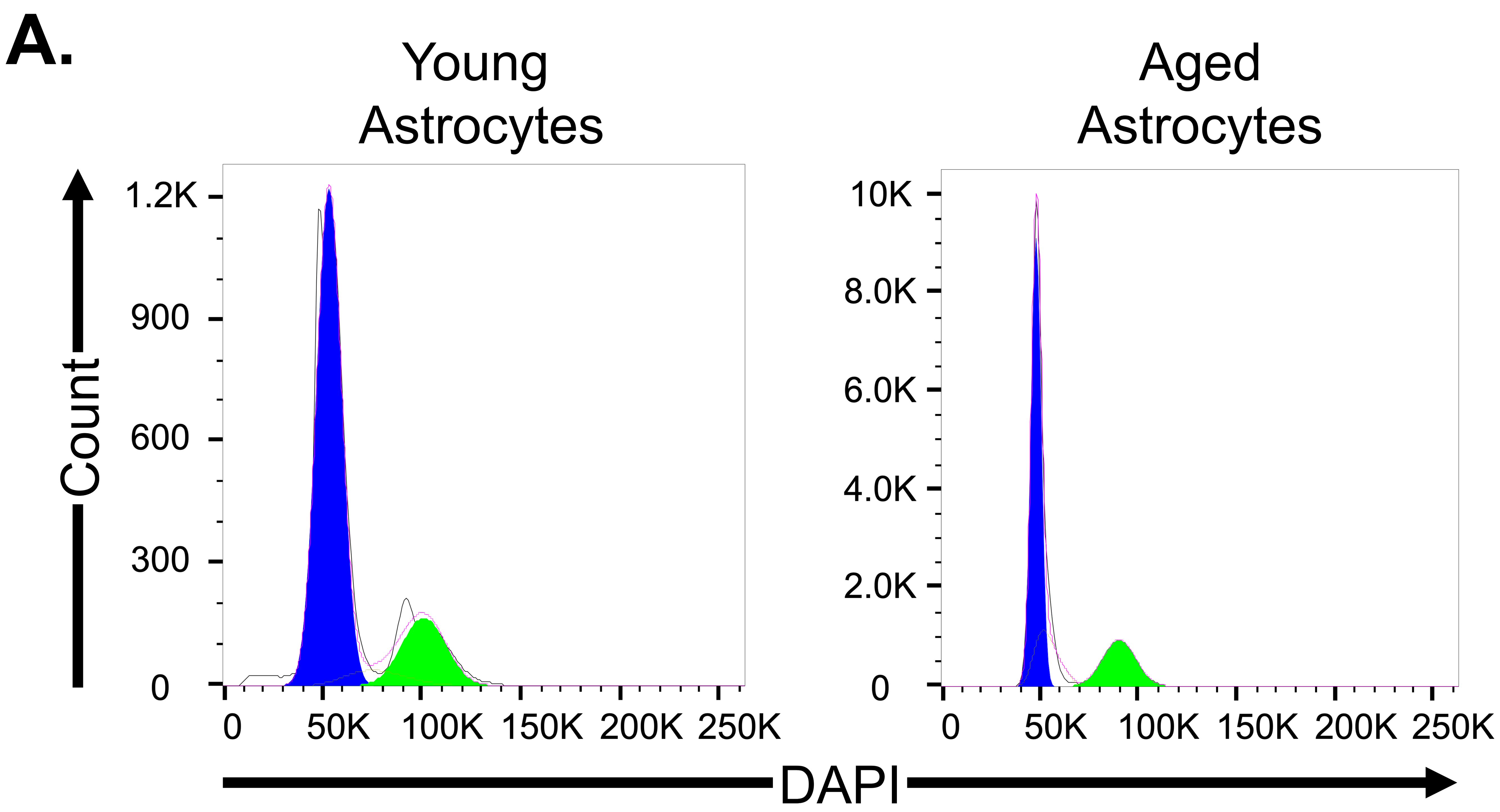

B.

	G_1	S	G_2
Young	76.81%	13.75%	8.95%
Aged	85.79%	5.61%	9.44%

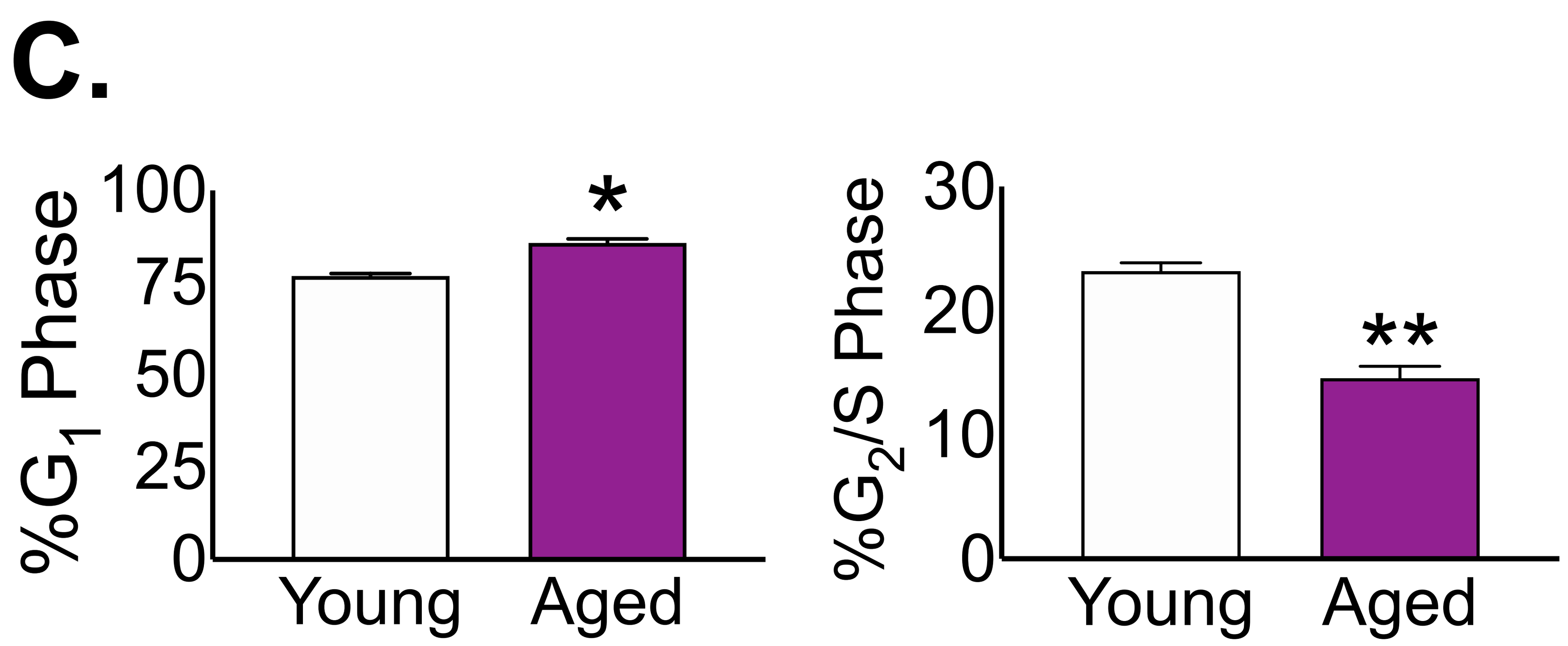

Supplementary Figure 2. Aged astrocytes exhibit cell cycle arrest. (**A**) Cell cycle analysis of young and aged astrocytes. (**B**) Average percentage of cells in each stage of cell cycle (n = 3) (**C**) Quantification of percentage of cells in each phase of cell cycle (n = 3) (**C**) **P* < 0.05, ***P* < 0.01, Student's t-test with Welch's correction. Values are expressed as mean ± SEM.

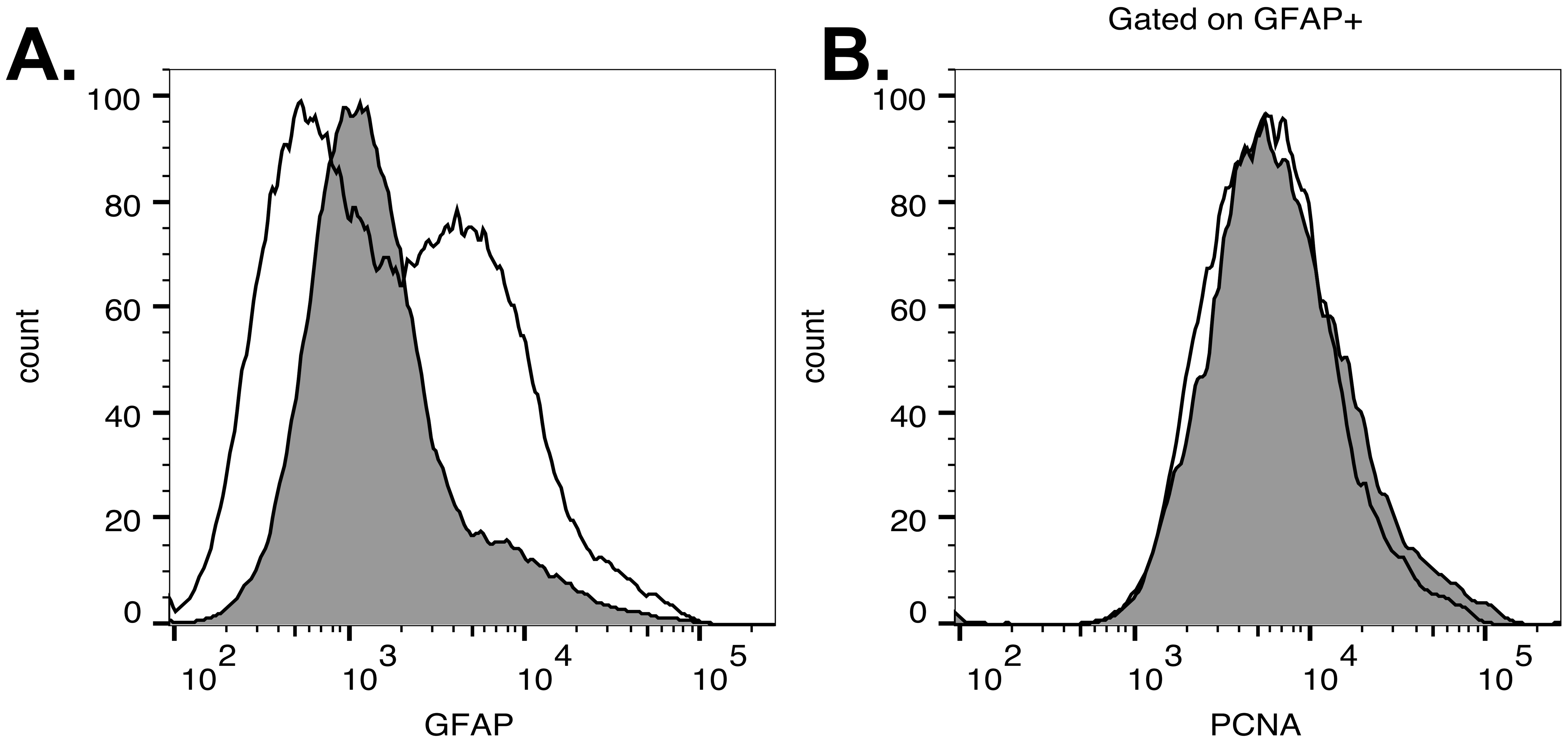

Supplementary Figure 3. Analysis of cellular proliferation in young and aged astrocyte cultures. Flow cytometry was peformed on young (<12 weeks) and aged (>16 weeks) cultures of primary astrocytes using GFAP and proliferating cell nuclear antigen (PCNA). (A) Aged cutures (white plots) show a bimodal distribution of proliferating GFAP+ cells, while (B) younger and older cultures exhibit a single overlapping profiles of PCNA+ cells.

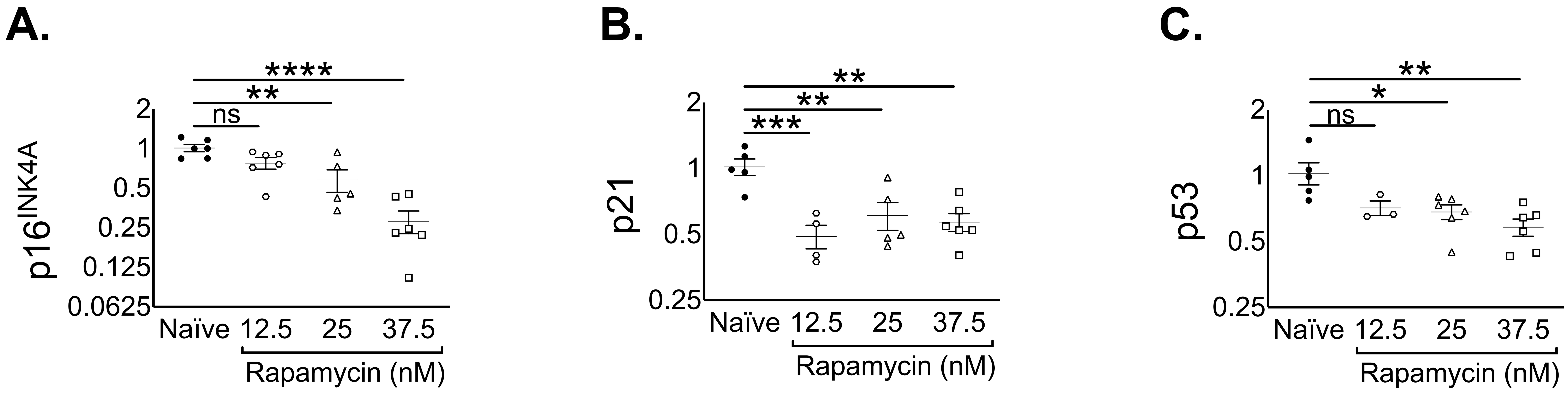

Supplementary Figure 4. Cellular senescence phenotype of aged astrocytes is reversed by treatment with rapamycin. mRNA expression analysis of aged astrocytes treated for 72 h (once/day) with increasing doses of rapamycin revealed reduced (**A**) p16^{INK4A}, (**B**) p21, and (**C**) p53 expression. Significance as indicated where: (**A**) ** *P* < 0.01; ****, *P* < 0.0001, one-way ANOVA, Dunnet's multiple comparisons test. (**B**) ** *P* < 0.01; ***, *P* < 0.001, one-way ANOVA, Dunnet's multiple comparisons test. (**C**) * *P* < 0.05; ** *P* < 0.01, one-way ANOVA, Dunnet's multiple comparisons test. Values are expressed as mean ± SEM.

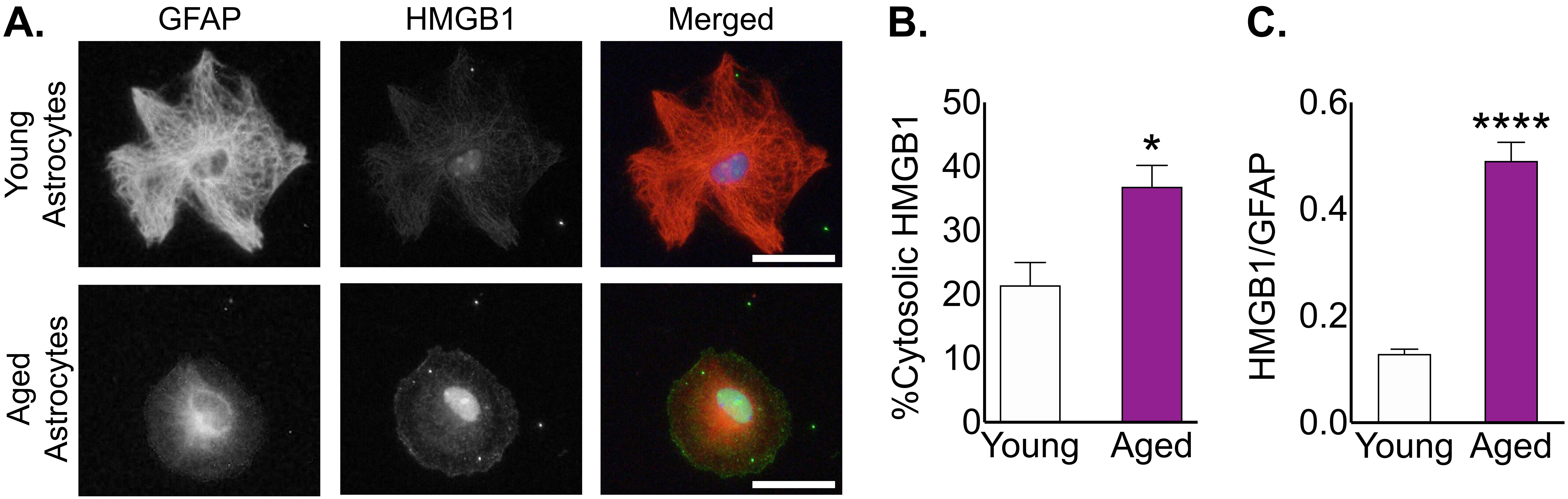

Supplementary Figure 5. Long-term culturing of aged astrocytes results in HMGB1 relocalization. (**A**) Representative immunocytochemistry of HMGB1 expression in young and aged astrocytes. Scale bar, 20 μm. (**B**) Quantification of percentage of cytosolic HMGB1 in young and aged astrocytes. (**C**) Quantification of HMGB1 and GFAP co-localization in individual cells in young and aged astrocytes. (**C**) **P* < 0.05, ****P* < 0.001, Student's t-test with Welch's correction. Values are expressed as mean ± SEM.

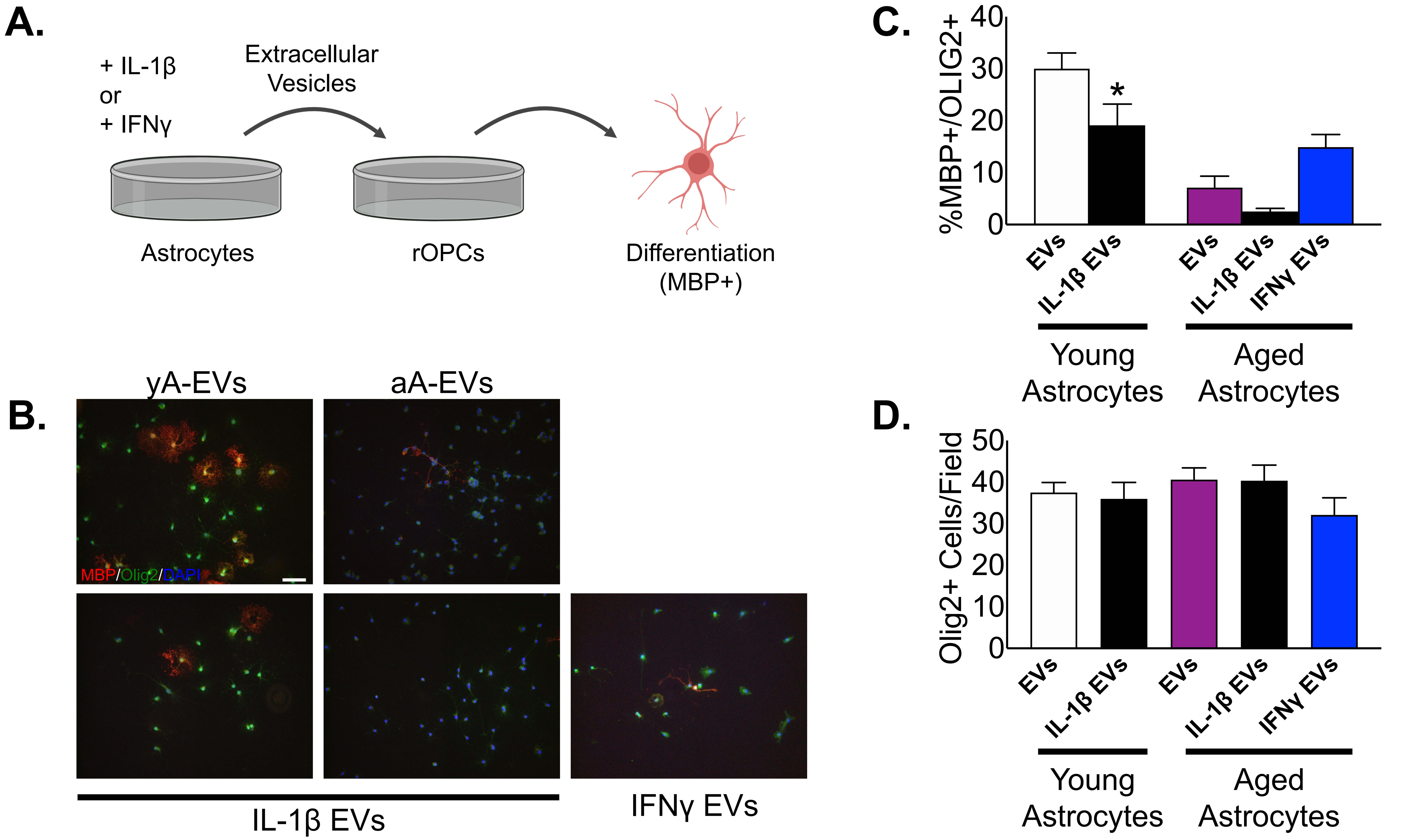

Supplementary Figure 6. Cytokine pre-treatment of young astrocytes impacts EV function. (**A**) Experimental design to test whether pre-treatment of young astrocytes with IL-1β and aged astrocytes with IL-1β or IFNγ effected the ability of EVs to promote OPC maturation (rOPCs, rat OPCs). ACM from cytokine-treated astrocytes was collected after 48 h, EVs isolated and applied to rOPCs. Differentiation of rOPCs was assayed after 48 h. (**B**) Representative images of mature oligodendrocytes (MBP+/OLIG2+) resulting from IL-1β treated young and IL-1β or IFNγ treated aged EVs. (**C**) Quantification of OL maturation following EV treatment from pre-treated young and aged astrocytes. (**D**) No differences in the number of OLIG2+ cells were observed from the varying treatments on the rOPCs. yA-EVs = young astrocyte EVs; aA-EVs = aged astrocyte EVs. *$P < 0.05$, one-way ANOVA, Tukey's multiple comparisons test). Values are expressed as mean ± SEM. Image of oligodendrocyte in Panel A from BioRender.com.

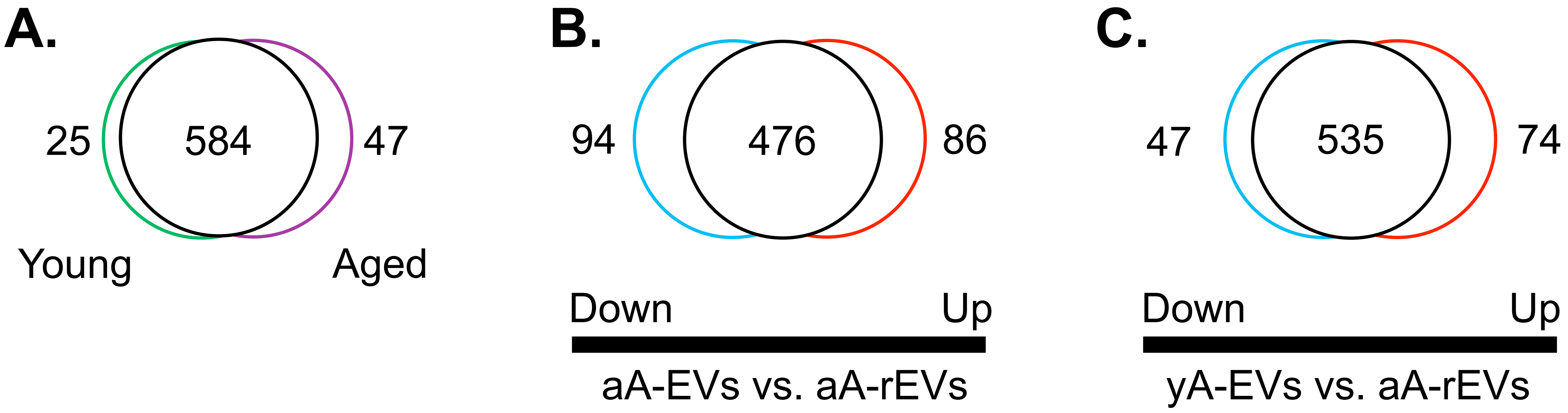

Supplementary Figure 7. Venn diagrams depicting the differential enrichment of peptides in EVs from young, aged and rapamycin-treated aged astrocyte cultures. (A) Young vs. Aged, (B) Aged astrocytes (aA) EVs vs. rapa-treated aged astrocytes (aA-rEVs), and (C) young astrocytes (yA) EVs vs rapa-treated aged astrocytes. Numbers within each finite set reflect the number of unique proteins identified for each condition. In (B) and (C), numbers reflect the direction of change, indicating the more (up) and less (down) abundant proteins unique to that comparison.

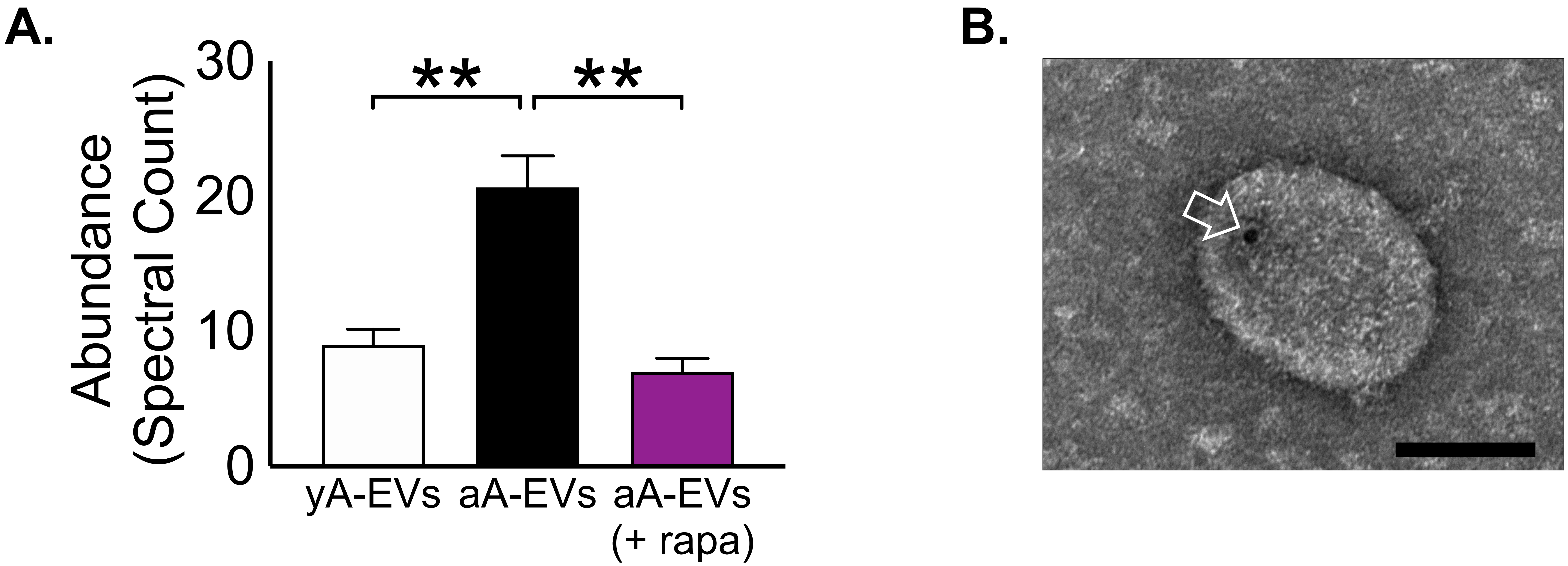

Supplementary Figure 8. Increased release of EV-associated HMGB1. (**A**) Quantification of spectral count abundance of HMGB1 on EVs from naïve young, naïve aged, and rapamycin treated astrocytes HMGB1. (**B**) Electron micrograph of astrocyte-derived EV from ACM verified by immunogold electron microscopy against HMGB1. White arrowhead indicates 10 nm HGMB1 gold particle. Scale bar, 100 nm. (**A**) $^{**}P < 0.01$, one-way ANOVA, Tukey's multiple comparisons test). Values are expressed as mean ± SEM.

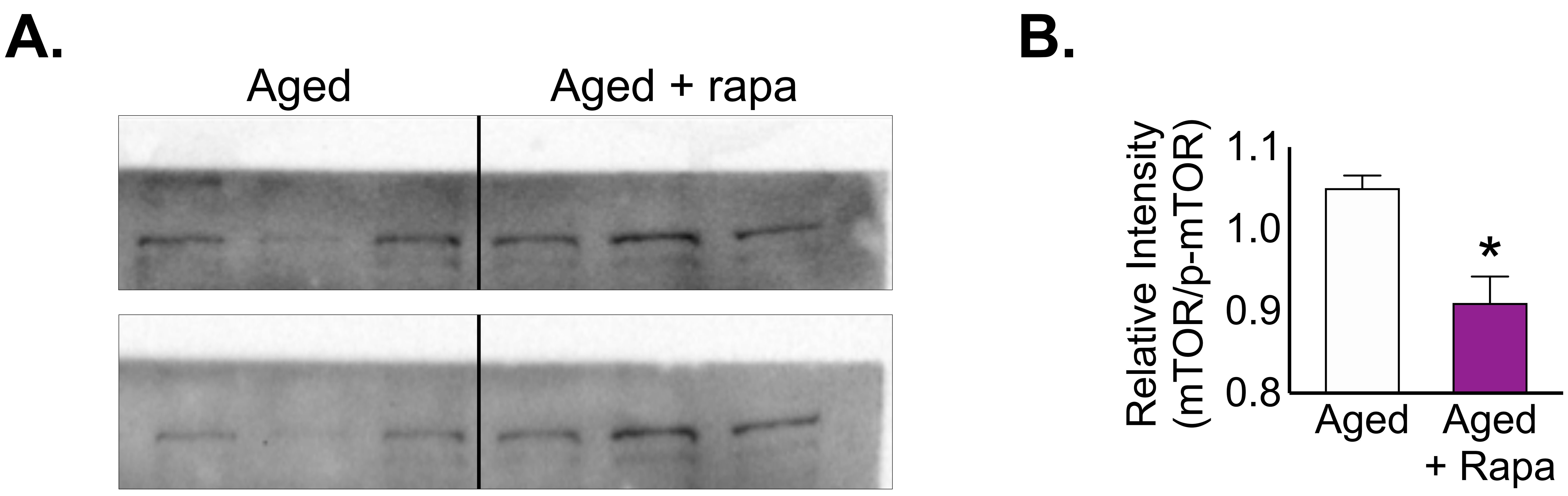

Supplementary Figure 9. Rapamycin Treatment reduced mTOR activation (**A**) Western blot of phospho-mTOR (top) and mTOR (bottom) in naïve and rapamycin-treated aged astrocytes (**B**) immunoblot analysis mTOR activation following rapamycin treatment

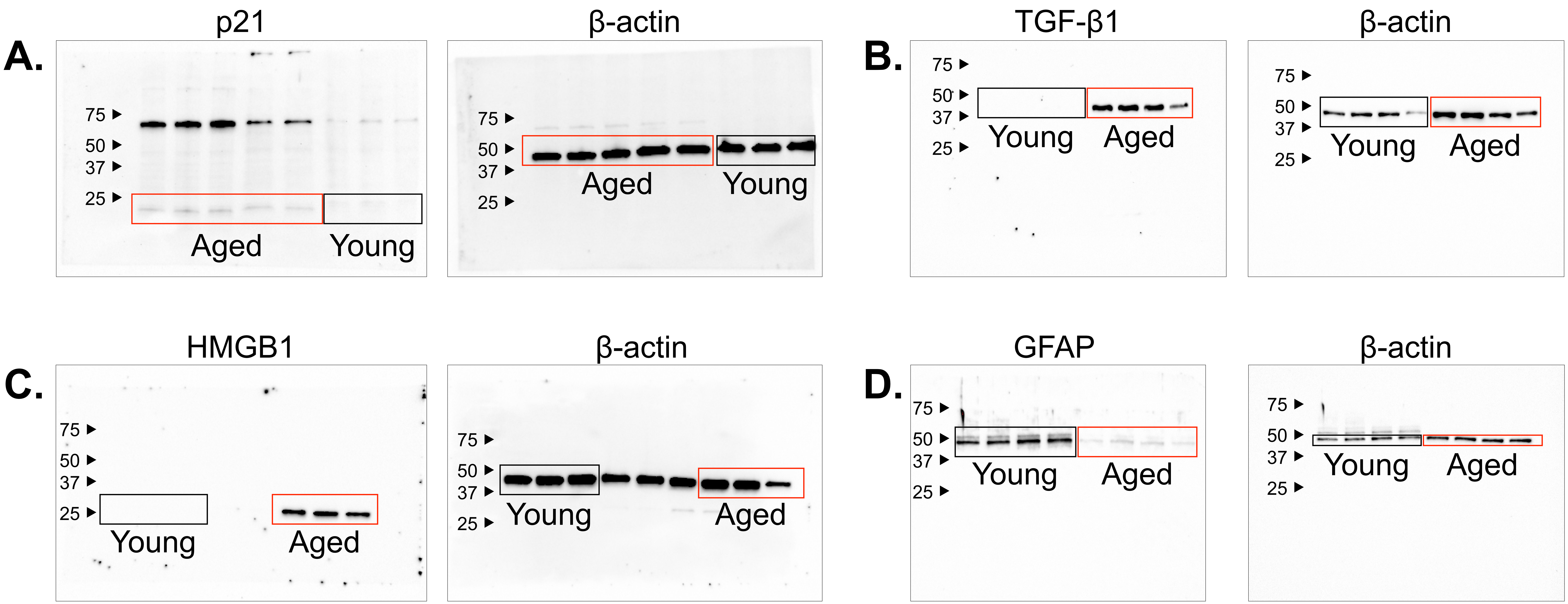

Supplementary Figure 10. Western blots shown in A-D are presented in panels C-F in Fig. 1. Analyses of (**A**) p21, (**B**) TGFB1 (**C**) HMGB1, and (**D**) the intermediate filament protein GFAP from young and aged astrocyte cell lysates, as indicated in highlighted boxes for each blot. Densitometry (a.u.) for each factor was used to determine expression relative to β-actin from each blot as shown. Blot images were captured at the same time using a BioRad ChemiDoc system. Each blot includes samples from each experimental group (ad indicated in legend of Fig. 1) and representative images are not modified from raw images captured using the gel imaging system.

Supplementary Table 1. qPCR Primer Sequences

Gene	Forward Sequence (5'-3')	Reverse Sequence (5'-3')
p16^{INK4A}	TACCCCGATTCAGGTGATGATG	TAGCTCTGCTCTTGGGATTGG
p21	AACATCTCAGGGCCGAAA	TGCGCTTGGAGTGATAGAAA
p53	CGACTACAGTTAGGGGGCAC	ATGGCAGTCATCCAGTCTTCG
Il6	TGTGCAATGGCAATTCTGAT	CTCTGAAGGACTCTGGCTTTG
Timp1	CATGGAAAGCCTCTGTGGATATG	AAGCTGCAGGCACTGATGTG
Mmp3	TAGAAATGGCAGCATCGATCTTC	GGAAATCAGTTCTGGGCTATACGA

Supplementary Table 2. Antibodies and Dilutions

Target	Dilution	Method	Company	Product #
DAPI	1:1000	ICC	Millipore-Sigma	268298
GFAP	1:1000	Western blot	Novus Biologicals	NB300-141
GFAP	1:10	Immunogold	Millipore-Sigma	AB5541
GFAP-Cy3	1:500	ICC	Millipore-Sigma	C9205
HMGB1	1:500	ICC / Western blot / Immunogold	BioLegend	651402
IBA-1	1:1000	ICC	WAKO	019-19741
MBP	1:500	ICC	Millipore-Sigma	MAB386
mTOR	1:1000	Western blot	Cell Signaling	MAB2983
OLIG2	1:500	ICC	Millipore-Sigma	AB9610
phospho-mTOR	1:1000	Western blot	Cell Signaling	MAB2974
$p16^{INK4A}$	1:500	ICC	ThermoFisher	MA5-17142
p21	1:500	ICC / Western blot	BD Biosciences	556430
TGF-β	1:1000	Western blot	BD Pharmingen	555052
TSG101	1:10	Immunogold	GeneTex	GTX63630
